# Leukaemia and lymphoma radiation risk models from atomic bomb survivors (1950–2001) suitable for lifetime risk calculations

**DOI:** 10.1007/s00411-026-01242-7

**Published:** 2026-08-10

**Authors:** Janine Becker, Felix Heinzl, Maria Schnelzer, Peter Scholz-Kreisel, Munechika Misumi, Hiromi Sugiyama, Kyoji Furukawa, Markus Eidemüller

**Affiliations:** 1 Federal Office for Radiation Protection, Oberschleißheim, Germany; 2 Radiation Effects Research Foundation, Hiroshima and Nagasaki, Japan; 3 Biostatistics Center, Kurume University, Kurume, Japan

**Keywords:** Radiation risk, Leukaemia risk models, Multi-model inference, Risk factors, ProZES software, Life Span Study, Leukaemia, Lymphoma

## Abstract

This paper updates radiation risk models for leukaemia and lymphoma incidence that can be applied to a variety of adult exposure scenarios and ages of onset of leukaemia and lymphoma, derived from the Life Span Study of atomic bomb survivors of Hiroshima and Nagasaki. Models were developed for four groups of haematological malignancies: acute lymphoblastic leukaemia (ALL), acute myeloblastic leukaemia (AML), chronic myeloid leukaemia (CML), and Hodgkin and non-Hodgkin lymphoma (LYM). Multi-model inference methodology was applied for all groups to construct flexible radiation risk models. The models revealed complex radiation risk patterns with strong dependencies on dose, age, and sex. The ALL group showed high radio-sensitivity with risk decreasing strongly with attained age. The AML group demonstrated a strong non-linear dose response requiring special considerations for fractionated or protracted exposures. The CML model showed a strong dependence on time since exposure. Excess relative risk for the lymphoma group was smaller than other groups and displayed significant sex differences. The developed models for leukaemia and lymphoma provide robust risk predictions for a large variety of adult exposure scenarios with ionising radiation. Multi-model inference proved to be an important tool in the construction of such flexible risk models. The models can be safely used in risk assessment tools or for lifetime risk evaluations.

**Supplementary Information:**

The online version contains supplementary material available at https://doi.org/10.1007/s00411-026-01242-7.

## Introduction

Radiation risk assessment for haematological diseases is highly challenging. While there is strong evidence of significantly increased leukaemia risk after exposure to low and high doses of ionising radiation (UNSCEAR 2006), the magnitude of risk as well as age and sex patterns are not well understood. The cohort of atomic bomb survivors of Hiroshima and Nagasaki (Life Span Study, LSS) revealed strong differences among subtypes of leukaemia (Hsu et al. 2013). Strong variations among leukaemia subtypes were also observed in worker studies (Leuraud et al. 2015, 2024).

The use of a single ‘all combined leukaemia’ model for risk projections thus appears problematic. Populations show different baseline rates and a different relative share of leukaemia subtypes. It is unclear how the non-linear dose dependence of a combined leukaemia model from the LSS could be used for protracted exposures in a reliable way. Similarly, the strong differences of age dependencies among the leukaemia subtypes require special considerations when applying the models to exposure patterns different from the LSS.

Constructing risk models for leukaemia and lymphoma presents a challenge that required careful consideration during the development of the software ProZES (BfS 2024). The program calculates the assigned share, which is the probability that a given cancer was caused by a preceding radiation exposure. ProZES is used in Germany for compensation claims of cancer after occupational exposure to ionising radiation. Therefore, the software has to provide robust and reliable estimations of the assigned share, also for fractionated and protracted exposures, and for all possible (adult) exposure ages and ages of onset of leukaemia (Ulanowski et al. 2020). The ProZES risk models were developed together with an international expert group and approved by the German Commission on Radiological Protection (SSK) and the German Federal Office for Radiation Protection (BfS).

The ProZES leukaemia and lymphoma models consist of 4 groups, mainly including acute lymphoblastic leukaemia (ALL), acute myeloblastic leukaemia (AML), chronic myeloid leukaemia (CML), and Hodgkin and non-Hodgkin lymphoma (LYM), as shown in Table 1. The risk models were previously derived from the LSS similar to Hsu et al. (2013) and published in Ulanowski et al. (2020). A main difference to Hsu et al. (2013) was the systematic use of multi-model inference, where a combination of models is used for construction of the final group models, as discussed below. Also, in ProZES the method of twin models was introduced for safe extrapolations towards low doses and dose rates for models with non-linear dose responses.

After first practical experience it turned out that the ProZES models (Ulanowski et al. 2020) did not provide plausible risk estimates for some possible exposure scenarios in occupational settings, e.g. for short latency times and young ages. This prompted a re-evaluation of the leukaemia and lymphoma models. Here we report on these updated models.

## Methodology

We shortly describe some concepts of particular relevance for the risk models. A detailed description of all methodological aspects is given in Ulanowski et al. (2020). The main text presents the radiation-related risk models with central parameter values. The supplementary file contains the complete models with parameter uncertainties and covariance matrices.

### Data base and grouping

The leukaemia and lymphoma risk models were derived from the LSS cancer incidence data. The analysis made use of the ‘lsshempy.csv’ dataset from the Radiation Effects Research Foundation (https://www.rerf.or.jp/en/library/data-en/lsshempy). It is an aggregated person-year table corresponding to the data used by Hsu et al. (2013), which included 113,011 LSS cohort members with follow-up from 1950 to 2001. The dataset included variables of characteristics of subjects (i.e., city, sex, ground distance from the hypocentre, category of distance from the hypocentre, age at the time of bombing (15 categories), attained age (18 categories), exposure information (i.e., DS02R1 weighted absorbed bone marrow dose (mGy) (gamma plus 10 times neutron; 23 categories), and outcome-related information (i.e., count of event, leukaemia and lymphoma groups, calendar time (14 categories), person-years, and person years adjusted for migration probabilities. The adjustment was necessary because cancer information was not available for LSS members who had migrated out of the registry catchment area. Migration probabilities were estimated from in- and out-migration patterns among Adult Health Study participants, a sub-cohort of the LSS. Models were developed for 4 groups of leukaemia and lymphoma following Ulanowski et al. (2020). Table 1 shows the definitions of the groups and the number of cases. CLL was included in the lymphoma group LYM with 10 observed cases in the LSS. While the overall grouping is similar to Hsu et al. (2013), there are also some differences. For example, Hsu et al. performed a separate analysis for non-Hodgkin and Hodgkin’s lymphoma, and also made an independent analysis for CLL.

**Table 1:** Leukaemia and lymphoma groups, ICD-10 diagnosis and number of cases in the LSS incidence study with follow-up 1950–2001

Model^a^	No.	Subtype	ICD10-Code(s)^b^
**ALL**	43	Acute lymphoblastic leukaemia (ALL)	C91.0
		B-cell type prolymphocytic leukaemia	C91.3
		Prolymphocytic leukaemia of the T-cell type	C91.6
		Other lymphoid leukaemia	C91.7
		Mature B-cell leukaemia Burkitt-type	C91.8
		Lymphoid leukaemia, unspecified	C91.9
**AML**	176	Acute myeloid leukaemia (AML)	C92.0
		Atypical chronic myeloid leukaemia	C92.2
		Myeloid sarcoma	C92.3
		Acute promyelocytic leukaemia	C92.4
		Acute myelomonocytic leukaemia	C92.5
		Acute myeloid leukaemia with 11q23-abnormality	C92.6
		Other myeloid leukaemia	C92.7
		Acute myeloid leukaemia with multilineage dysplasia	C92.8
		Myeloid leukaemia, unspecified	C92.9
		Monocytic leukaemia	C93
		Other leukaemia of designated cell type	C94
		Leukaemia of unspecified cell type	C95
		Other or unspecified malignant neoplasms of lymphoid, hematopoietic and related tissues	C96
**CML**	75	Chronic myeloid leukaemia (CML)	C92.1
**LYM**	449	Hodgkin‘s lymphoma	C81
		Non-Hodgkin lymphoma	C82, C83, C84, C85, C86
		Malignant immunoproliferative diseases	C88
		Chronic lymphocytic leukaemia (CLL)	C91.1
		Hairy cell leukaemia	C91.4

^a^: The groups correspond to the groups by Hsu et al. (2013) with ALL = *alltot*, AML = *amltot*, CML = *cml*, LYM = *nhl+hl+clltot *in the lsshempy dataset

^b^: Contemporary cancer classification is more detailed than in previous times. Table 1 suggests which ICD-10 codes are most closely related to each specific risk model. In practical application of ProZES, for a cancer with given ICD code the most appropriate risk model should be selected

### Risk models

The incidence rates are represented either as models of excess relative risk (ERR) or excess absolute risk (EAR), given by

1 $$\:\boldsymbol{\lambda\:}={\boldsymbol{\lambda\:}}_{0}\left(1+\mathbf{E}\mathbf{R}\mathbf{R}\right)={\boldsymbol{\lambda\:}}_{0}+\mathbf{E}\mathbf{A}\mathbf{R}$$

2 $$\:{\boldsymbol{\lambda\:}}_{0}=\boldsymbol{f}\left({\boldsymbol{\upbeta\:}}_{\boldsymbol{b}}\right|\boldsymbol{s},\boldsymbol{a},\boldsymbol{b})$$

Here, $\:{\lambda\:}_{0}$ is the baseline incidence rate in the absence of radiation depending on a function *f* of sex *s*, attained age *a* and birth year *b*, and $\:\lambda\:$ is the total incidence rate after radiation. The $\:{\beta\:}_{b}$’s represent the fit parameters of the baseline. A generic form of the baseline is given by $\:{\lambda\:}_{0}=\mathrm{e}\mathrm{x}\mathrm{p}({\beta\:}_{1}+{\beta\:}_{2}\cdot\:\mathrm{l}\mathrm{n}(a/70)+{\beta\:}_{3}\cdot\:{\mathrm{l}\mathrm{n}}^{2}(a/70)+{\beta\:}_{4}\cdot$ *(b-1915)/10)*, the $\:\beta\:$‘s can also be sex dependent. Specific models for the different endpoints can be found in the supplementary file. The radiation risk can be separated into a radiation-related part depending on time-lagged total dose *D*, and a part depending on sex, attained age, age at exposure *e*, or time since exposure *tse*:

3 $$\:\left.\begin{array}{c}\mathbf{E}\mathbf{R}\mathbf{R}\\\:\mathbf{E}\mathbf{A}\mathbf{R}\end{array}\right\}=\boldsymbol{r}\left({\boldsymbol{\upbeta\:}}_{\boldsymbol{r}}|\boldsymbol{D}\right)\:\boldsymbol{g}\left({\boldsymbol{\upbeta\:}}_{\mathbf{g}}|\boldsymbol{s},\boldsymbol{a},\boldsymbol{e},\:\boldsymbol{t}\boldsymbol{s}\boldsymbol{e}\right).$$

The $\:{\beta\:}_{r}$’s represent the parameters related to radiation and $\:{\beta\:}_{g}$’s represent those of effect modifiers. In order to obtain robust fits for these models we tested several model variants including additional effect modifiers. If the inclusion of a parameter didn’t lead to a significant reduction in deviance, we excluded it from the final model in favour of a lower number of parameters. Age at exposure, time since exposure and attained age were tested for all models. The parameters and uncertainty bounds were obtained by a maximum likelihood fit, keeping parameters which are significant at the 95% CI level (Ulanowski et al. 2020).

The LSS data provide the organ equivalent dose to the bone marrow in Gray (Gy). Various dose functions $\:r\left({\boldsymbol{\upbeta\:}}_{\boldsymbol{r}}\right|D)$ were tested, including linear, linear-quadratic, quadratic, and spline functions.

As can be seen from Eq. (1), the radiation part of $\:\lambda\:$ from the ERR model is multiplied by the baseline rate $\:{\lambda\:}_{0}$. Since the baseline rate is strongly dependent on age, fitting ERR and EAR models usually results in different age dependencies for the two types of models. The models were fitted with Poisson regression using the programming language R with the optimization routine optimx^1^. The final models were selected by minimizing the Akaike Information Criterion AIC. Since the follow-up started in year 1950, the data contain no information on a latency time between exposure and leukaemia risk within the first 5 years after exposure, and the models are independent of an assumed latency. For presentation of the results, we assumed a latency time of 2 years following UNSCEAR (2006), which is also consistent with a biological model of leukaemia growth data (Little et al. 2024).

### Multi-Model Inference (MMI)

With the MMI method each of the leukaemia groups is described not by a single model, but by two or more models. A distribution of ERR/EAR values is then calculated from the different individual risk models using their corresponding weights (Walsh and Kaiser 2011). The use of MMI reduces the dependence of the risk estimates on the choice of one particular model and provides a more realistic assessment of uncertainties, including also uncertainties from model selection. Usually, the model weights are determined by the quality of fit to the data, given by the AIC. However, the development of robust risk models for some leukaemia groups additionally required a weighting based on expert judgment, as discussed below in more depth for CML.

### Twin models

Risk models with strong non-linear dose responses can lead to problematic extrapolations when applying the models to fractionated or protracted exposures, e.g. for the statistically preferred pure quadratic dose-response model for AML. An example is presented in the Discussion. Thus, for any model with a non-linear dose response, an additional ‘twin’ model with linear low-dose response was fitted to the data. For the low dose region until the first intersection point of both risk functions, linear and non-linear, the linear model is selected. From the intersection point onwards to higher doses usual MMI with both models is applied. The dose response of the combined twin model guarantees safe extrapolation to low doses and prevents potential strong underestimation of risk (Ulanowski et al. 2020).

## Results

Detailed information on the risk models can be found in the supplementary file.

### Group ALL

The ALL group has a strong dose response. Among various ERR and EAR models, two EAR models provided the best fit to the data (lowest AIC), with 77.4% weight for the linear model and 22.6% weight for the model with quadratic dose response. Both models form a twin pair. The EAR is given per 10^4^ person years, total time-lagged dose D is given in Gy.

4 $$\:{\mathbf{E}\mathbf{A}\mathbf{R}}_{\boldsymbol{L}\boldsymbol{N}\boldsymbol{T}}=0.27\:\boldsymbol{D}\:\mathbf{e}\mathbf{x}\mathbf{p}\left[-1.79\mathbf{ln}\left(\frac{\boldsymbol{a}}{70}\right)-0.93\:\boldsymbol{f}\right]$$

5 $$\:{\mathbf{E}\mathbf{A}\mathbf{R}}_{\boldsymbol{Q}\boldsymbol{D}\boldsymbol{R}}=0.17{\:\boldsymbol{D}}^{2}\:\mathbf{exp}\left[-1.87\mathbf{ln}\left(\frac{\boldsymbol{a}}{70}\right)-0.91\:\boldsymbol{f}\right]$$

Due to the form of the baseline, the EAR and ERR formulations are mathematically equivalent under reparameterization (see Supplement). However, the age parameters in the ERR formulation are substantially more correlated than in the EAR formulation, which inflates the uncertainty of the estimated risk parameters. The EAR models were therefore selected as preferred.

**Fig. 1:**
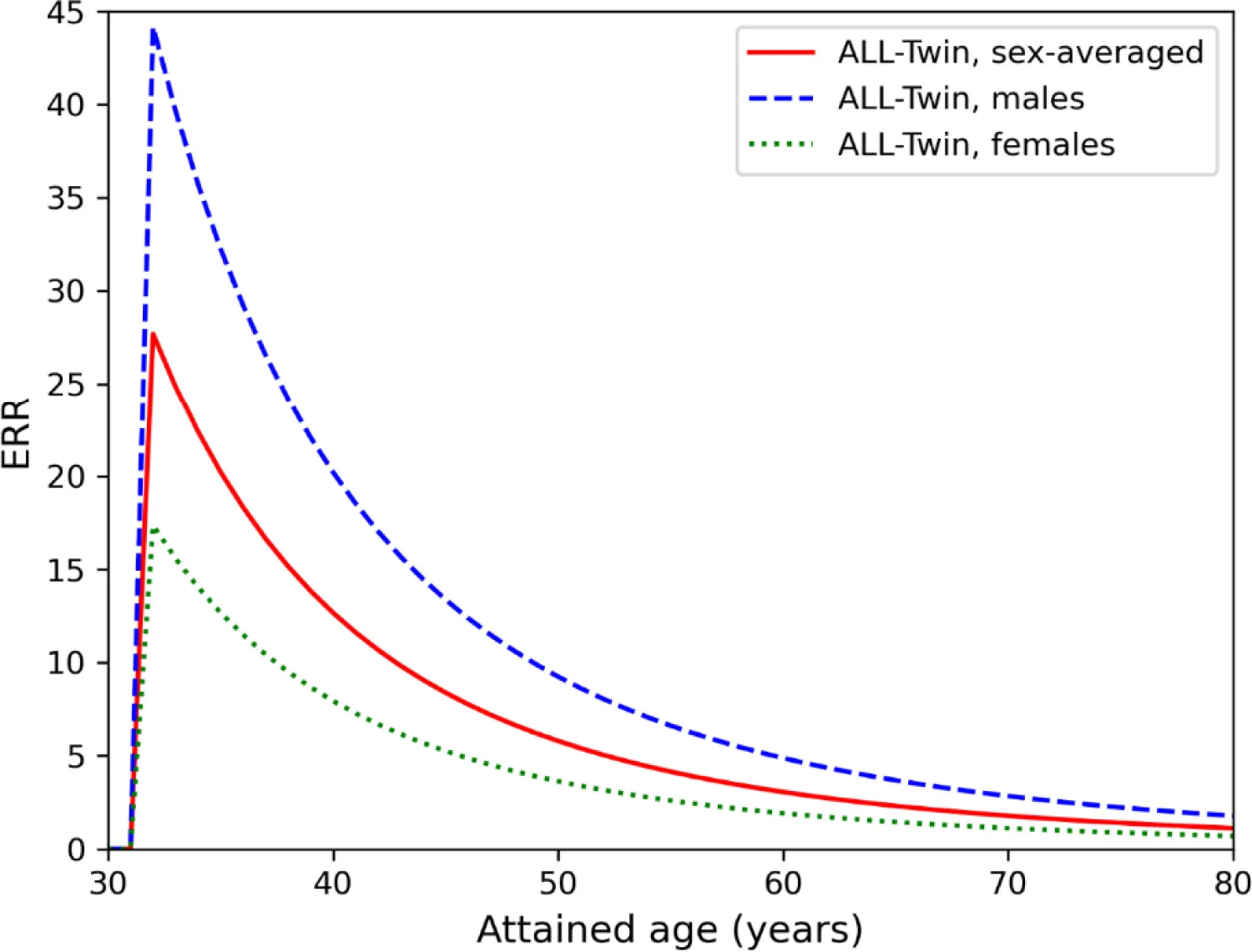
Dependence of the ERR on attained age for the ALL group for an exposure of 1 Gy at age 30. The ERR shows a strong decrease with increasing attained age. Risk for males is higher than for females. A minimum latency period of 2 years was assumed

Figure 1 shows the ERR as a function of attained age for an exposure of 1 Gy at age 30. The ERR value is obtained by dividing the EAR value by the baseline of the LSS. A minimum latency time of 2 years was assumed, so the predicted risk vanishes during the first 2 years. The risk is sex dependent with lower risk for females (* f=1* for females, * f=0* for males). The relative risk has a strong dependence on attained age, and the ERR becomes large at younger ages.

### Group AML

The AML group has a strong non-linear dose response. The model with largest weight is an EAR model with pure quadratic dose response (EAR-QDR, 50.4% weight). It forms a twin pair with a two-line-spline dose response model (EAR-TLS, 34.3%). Similarly, the ERR-QDR model (9.3%) forms a twin pair with the ERR-TLS model (5.9%).

6 $$\:{\mathbf{E}\mathbf{A}\mathbf{R}}_{\boldsymbol{Q}\boldsymbol{D}\boldsymbol{R}}=\mathbf{exp}\left[2.72\:\mathbf{l}\mathbf{n}\frac{\boldsymbol{a}}{70}+1.48\:\mathbf{l}\mathbf{n}^2\left(\frac{\boldsymbol{a}}{70}\right)\right]\cdot\:1.23\:{\boldsymbol{D}}^{2}$$

7 $$\begin{array}{l}\:{\bf{EA}}{{\bf{R}}_{TLS}} = {\bf{exp}}\left[ {2.51\:{\bf{ln}}\frac{a}{{70}} + 1.38\:{\bf{l}}{{\bf{n}}^2}\left( {\frac{a}{{70}}} \right)} \right]\\ \cdot \:\left\{ {\begin{array}{*{20}{c}}{0.8\:D}\\{\:0.8\:\tau \: + 4.24\:\left( {D - \tau \:} \right)}\end{array}} \right.\begin{array}{*{20}{c}}{D < \tau \:}\\{\:D > \tau \:}\end{array}\end{array}$$

8 $$\:{\mathbf{E}\mathbf{R}\mathbf{R}}_{\boldsymbol{Q}\boldsymbol{D}\boldsymbol{R}}=2.21{\:\boldsymbol{D}}^{2}$$

9 $$\:{\mathbf{E}\mathbf{R}\mathbf{R}}_{\boldsymbol{T}\boldsymbol{L}\boldsymbol{S}}=\left\{\begin{array}{c}1.33\:\boldsymbol{D}\\\:1.33\:\boldsymbol{\tau\:}+7.62\:(\boldsymbol{D}-\boldsymbol{\tau\:})\end{array}\right.\begin{array}{c}\:\boldsymbol{D}<\boldsymbol{\tau\:}\\\:\:\boldsymbol{D}>\boldsymbol{\tau\:}\end{array}$$

**Fig. 2:**
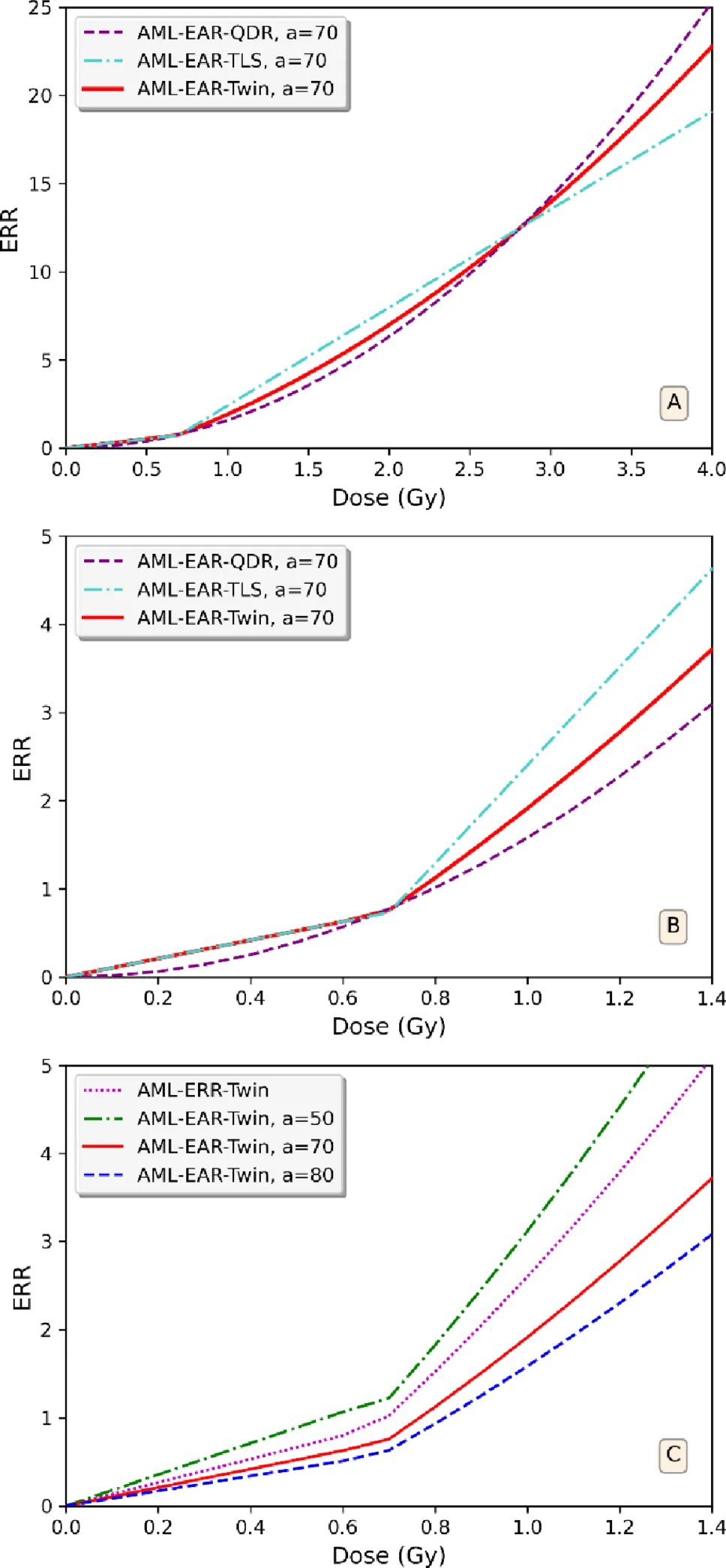
Dependence of the ERR on dose for the AML group. The ‘twin’ method was used to provide safe extrapolation towards low doses and protracted exposures. Panel A and B show the quadratic and two-line-spline EAR models together with the twin model for dose ranges until 4 Gy and 1.4 Gy, respectively. Panel C shows several EAR twin models for different attained age, the ERR twin model is independent of attained age

Figure 2 shows the dependence of ERR with dose for the AML group. Panel A and B show the sex-averaged EAR-QDR and EAR-TLS models with largest weights. The ERR value is obtained by dividing the EAR value by the baseline. The behaviour of the ERR models is similar and is not displayed for reasons of clarity. For the TLS model, risk increases with a smaller slope until $\:\tau\:=0.7$ Gy, and with larger slope for higher doses. As described in the supplementary file, the value of $\:\:\tau\:$ was obtained by sequentially increasing $\:\:\tau\:$ in 0.1 Gy steps and using maximum likelihood. For the twin model, usual multi-model inference is employed for doses larger than about 0.7 Gy, which here naturally separates the lower- and higher dose region. Below 0.7 Gy, the linear model is selected for the twin model if its ERR value is larger than the quadratic model. The EAR-Twin model in the figure shows the resulting risk function valid for the full dose range.

In panel B, which shows the lower dose range in more detail, it can be seen that the linear model is selected in the full lower dose range until about 0.7 Gy since in this dose range the quadratic model lies below the linear one. While the ERR model is independent of attained age, the ERR values of the EAR model depend on attained age. Panel C shows that ERR decreases with increasing attained age. Since the fit quality of the EAR models is better than that of the ERR models, it can be expected that the EAR models reflect better the age dependence of risk in the underlying LSS data. The final AML model with 84.7% weight for the EAR models and 15.3% weight for the ERR models is very close to the AML-EAR-Twin model in Fig. 2C. The introduction of the twin model strongly improves the plausibility of risk predictions for fractionated and protracted exposures, as discussed below.

### Group CML

In the LSS, radiation risk of CML has a very distinct age dependence compared to the other groups. It strongly depends on time since exposure (TSE), but has no significant additional dependence on attained age or age at exposure. The LSS data show very high radiation risk within the first 10 years of TSE, medium-high risk between 10 and 40 years, and no risk for more than 40 years of TSE. Correspondingly, the best fitting model is an ERR model linear in dose with a step-like dependence on TSE. However, the sole use of this model is very problematic for risk projections and its use in ProZES. Such strong steps in risk are biologically not plausible. Thus, a second model with exponential TSE dependence was fitted to the data. However, this model leads to even higher risk when extrapolated to short TSE. Furthermore, both models have the problem that they predict zero or almost vanishing risk for long TSE. The INWORKS study does not suggest a continuously decreasing risk with TSE (Daniels et al. 2017, 2024). Therefore, a third model without TSE dependence was fitted to the LSS data. This model gives risk estimates compatible with INWORKS (Leuraud et al. 2024), however, it does not reflect the TSE dependence observed in the LSS. A weighting of the models according to fit quality was not possible since basically only the step model would survive. Since it is difficult to give a preference for some of these arguments, it was decided to weight each model equally with 1/3. This compensates the potentially problematic behaviour of each single model and results in plausible risk estimates over the full TSE range.

10 $$\:{\mathbf{E}\mathbf{R}\mathbf{R}}_{\boldsymbol{S}\boldsymbol{t}\boldsymbol{e}\boldsymbol{p}}=\left\{\begin{array}{cc}40.7\:\boldsymbol{D}&\:\:\:0<\boldsymbol{t}\boldsymbol{s}\boldsymbol{e}<10\\\:5.7\:\boldsymbol{D}&\:10<\boldsymbol{t}\boldsymbol{s}\boldsymbol{e}<40\\\:0&\:40<\boldsymbol{t}\boldsymbol{s}\boldsymbol{e}\:\:\:\:\:\:\:\:\:\:\:\end{array}\right.$$

11 $$\:{\mathbf{E}\mathbf{R}\mathbf{R}}_{\boldsymbol{E}\boldsymbol{x}\boldsymbol{p}}=74.2\:\boldsymbol{D}\:{\boldsymbol{e}}^{-0.11\:\boldsymbol{t}\boldsymbol{s}\boldsymbol{e}}$$

12 $$\:{\mathbf{E}\mathbf{R}\mathbf{R}}_{\boldsymbol{C}\boldsymbol{o}\boldsymbol{n}\boldsymbol{s}\boldsymbol{t}}=8.2\:\boldsymbol{D}$$

**Fig. 3:**
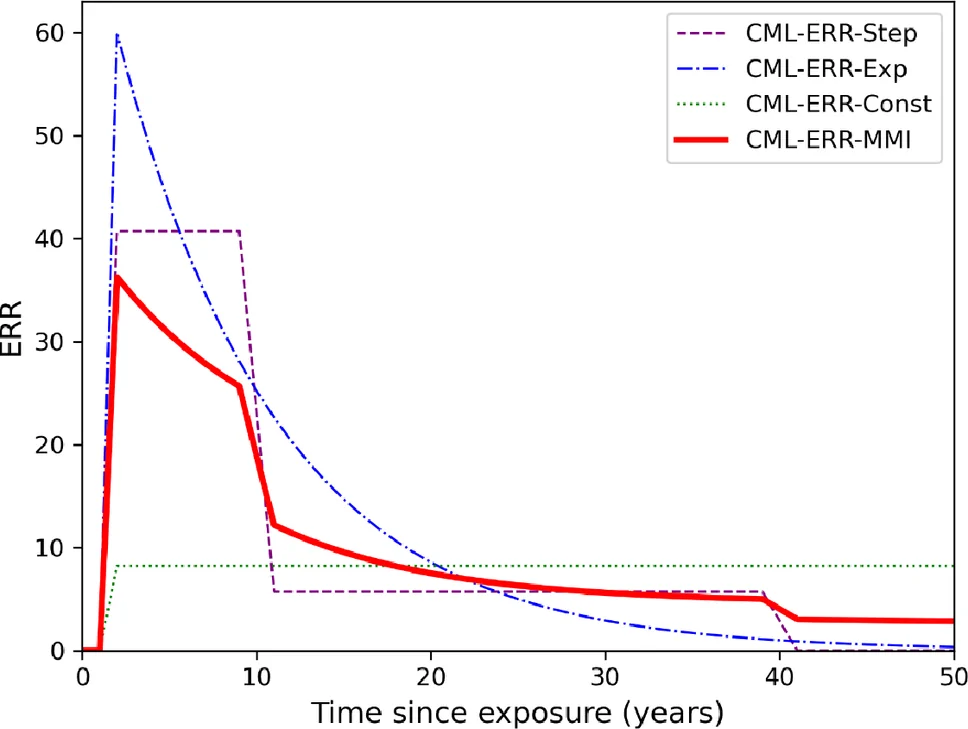
Dependence of ERR on time since exposure for the CML group for a dose of 1 Gy. MMI was used to construct a risk model, based on equal weighting of the three individual models, that reflects a compromise between LSS data, results from INWORKS, and biological plausibility, and can safely be used for all values of TSE. A minimum latency period of 2 years was assumed

The individual models and the combined MMI model are presented in Fig. 3. It can be seen that for short TSE the risk of the combined model is attenuated compared to the exponential and the step model. Between TSE of 10 and 40 years the ERR decreases, but not as strong as the exponential model. For longer TSE, the combined model still predicts a non-vanishing risk, whereas the exponential and the step model essentially give zero risk.

### Group LYM

The group LYM includes various lymphoma, most cases are non-Hodgkin lymphoma (402 cases). The baseline is the same for both sexes. Excess relative risk is generally lower than for the other groups. A separate fit for males and females results in significant radiation risk for males, but zero radiation risk for females. A joint fit for both sexes also results in positive radiation risk. It is biologically not plausible that males would be at risk for radiation-induced lymphoma, but not females. Therefore, to find a compromise between epidemiological evidence and biological plausibility, the following procedure was adopted with the help of MMI: 50% weight was assigned to the sex (male) specific risk model, and 50% weight to the joint risk model (Ulanowski et al. 2020). Within each of the two model sub-groups an AIC-based weighting was performed, resulting in one ERR-type (32.9% weight) and one EAR-type (17.1%) model for the joint set of models, and one ERR-type (19.0%) and one EAR-type (31.0%) model for the male-only model set. All models are linear in dose:

13 $$\:\mathbf{E}\mathbf{A}{\mathbf{R}}_{\boldsymbol{J}\boldsymbol{o}\boldsymbol{i}\boldsymbol{n}\boldsymbol{t}}=0.13\:\boldsymbol{D}$$

14 $$\:{\mathbf{E}\mathbf{R}\mathbf{R}}_{\boldsymbol{J}\boldsymbol{o}\boldsymbol{i}\boldsymbol{n}\mathbf{t}}=0.29\:\boldsymbol{D}$$

15 $$\:{\mathbf{E}\mathbf{A}\mathbf{R}}_{\boldsymbol{M}\boldsymbol{a}\boldsymbol{l}\boldsymbol{e}}=0.63\:\boldsymbol{D}$$

16 $$\:{\mathbf{E}\mathbf{R}\mathbf{R}}_{\boldsymbol{M}\boldsymbol{a}\boldsymbol{l}\boldsymbol{e}}=0.61\:\boldsymbol{D}$$

## Discussion

Leukaemia and lymphoma are serious health concerns after radiation exposure of workers, patients, or the general population. These diseases are highly radiosensitive and form an essential part in calculations of lifetime risk and the detriment (UNSCEAR 2019; ICRP 2007). Their risk models are highly challenging due to limited epidemiological data, non-linear dose responses, strong temporal effects, sex differences, and differences in population baseline rates. It is essential to include the age dependencies into the risk models (SSK 2023) as leukaemia is highly age-related.

This work re-evaluated the leukaemia and lymphoma models of Ulanowski et al. (2020). The focus was on developing robust risk models, i.e., models that provide plausible risk predictions for possible exposure scenarios mainly during a person’s working life. They provide safe extrapolations for low doses and protracted exposures. The presented models are implemented into the ProZES software and are used in calculations of the assigned share. The subtypes of haematological malignancies, separated into the groups ALL, AML, CML and LYM, have highly different risk characteristics in the LSS. The lymphoma group including non-Hodgkin and Hodgkin’s lymphoma has lower excess relative risk than the other groups. The number of cases for Hodgkin lymphoma alone is too small to obtain a significant fit for radiation risk. When excluded, the radiation risk parameters for non-Hodgkin lymphoma changes by up to 25%, which is within the parameter uncertainties (see Supplement).

Lymphoma risk for females in the LSS is zero whereas the risk for males is significant. No age dependence for lymphoma radiation risk is observed. ALL and related endpoints have a strong dose response, in particular at younger age, and the risk decreases strongly with increasing attained age.

The AML group is characterized by a markedly non-linear dose response, with the best risk model being purely quadratic in dose. Let us investigate the risk predictions of this highest-weighted EAR quadratic dose response model. A single exposure of 1 Gy at age 30 leads to an ERR at age 70 of $\:\mathrm{E}\mathrm{R}\mathrm{R}=1.23\cdot\:{10}^{-4}/(0.78\cdot\:{10}^{-4})=1.58$, where the numerator can be read off Eq. (6), and the denominator is the sex-averaged baseline hazard for birth year 1915 from the LSS as given in the supplementary file. If the exposure is separated into 10 fractions of 100 mGy each, the resulting risk is $\:10\times\:1.58\times\:{0.1}^{2}=0.158$, which is 10 times smaller than a single exposure. 100 fractions of 10 mGy would reduce the risk even further to an ERR of 0.0158. It is biologically implausible that a single exposure could lead to a substantial radiation risk, while fractionated exposures with the same total dose would result in almost no risk. Furthermore, the risk is not stable with changes in fractionation at low doses. This behaviour is likely an artefact of using a quadratic dose-response model, which inappropriately applies the curvature of risk observed at higher doses to lower doses. Introducing the twin model resolves this issue. With the EAR-Twin model, a single exposure of 1 Gy results in an ERR of 1.91. For 10 fractions of 100 mGy the risk is somewhat reduced to an ERR of 1.05, and 100 fractions of 10 mGy result in the same risk of 1.05. Thus, risk predictions remain stable with variations in fractionation. The recent INWORKS analysis reports an AML risk of ERR = 0.75 per Gray for protracted exposure among workers (Leuraud et al. 2024). This finding aligns well with the results from the twin model for fractionated exposures and becomes even closer when considering males only, with an ERR of 0.64 from the LSS twin model evaluated for fractionated exposures. Still, some caution is warranted due to the substantial uncertainty in the INWORKS AML result, but it does demonstrate the overall consistency of the twin approach.

CML has a strong dependence on time since exposure (TSE) with highest risk for short TSE, and lowest risk for long TSE. It is the group with the largest substantial change compared to Ulanowski et al. (2020). The group now consists of three models with different TSE dependencies. While each single model has significant shortcomings, it was possible to achieve reasonable risk predictions for all TSE by using MMI with equal weighting among the three CML models.

It is not clear whether the leukaemia models can be directly extrapolated to childhood exposure and risk. Biological development of childhood leukaemia can be different from adults. Recently, a large study evaluated haematological malignancies for children after CT scans (Bosch de Basea Gomez et al. 2023). Risk estimates were found to be higher than in the atomic bomb survivors. The recent study by Fujihara et al. (2022) analysed the incidence of lymphoid neoplasms in atomic bomb survivors using the updated WHO classification (Swerdlow et al. 2008). Our cohort data does not provide the level of subtype information required for such an analysis.

The models should not be directly used for therapeutic exposures, i.e., acute exposures substantially above 1 Gy. In Simonetto et al. (2021) a review of the literature on leukaemia dose response after therapeutic exposures resulted in a plateau of ERR at higher doses. The PASSOS software estimates risk of heart disease and second primary cancer after breast cancer radiotherapy, including leukaemia risk. The risk models of this work are included in the PASSOS software until acute exposures of 1 Gy, for higher local doses only the high-dose risk models are used (Simonetto et al. 2021).

An overarching property of all risk models presented here is the use of multi-model inference. It reduces the dependence on single models and allows to estimate model uncertainty. In addition, it is a powerful tool to include information that is external to the investigated cohort, i.e., model formulation can incorporate findings from other studies. Among the 310,000 nuclear workers of the INWORKS study 771 non-CLL leukaemia, 1146 non-Hodgkin lymphoma, and 122 Hodgkin lymphoma deaths were observed between 1944 and 2016. The average cumulative red bone marrow dose in the INWORKS study was 16.2 mGy, and about 40 radiation-induced non-CLL leukaemia deaths from a linear ERR model were estimated (Leuraud et al. 2024). For CML, the INWORKS study does not suggest a continuously decreasing risk with TSE, and finds strongly increased risk between 20 and 30 years of TSE, although the variations and uncertainties in the different time periods are large (Daniels et al. 2017, 2024). Therefore, a model with constant TSE dependence was added. The resulting combined model thus reflects temporal (TSE-related) patterns informed by both the LSS and INWORKS. Also, MMI was used to achieve a compromise between epidemiological evidence and biological plausibility for the sex differences in the LYM group.

A major limitation for leukaemia risk assessment is the relatively small number of cases, in particular for leukaemia subtypes. Compared to solid cancers, it is more difficult to separate the spontaneous and radiation-induced cases. Still, due to larger attributable fractions, radiation risks and age dependencies can be derived. Use of the models for risk projections requires the transfer of risk to other populations. It is unknown how reliable leukaemia and lymphoma risk from LSS members can be applied to modern non-Asian populations. Although the developed models provide consistency to INWORKS, transfer of risk between populations is an important area for future research.

In summary, the leukaemia and lymphoma models were constructed to provide plausible risk estimates for all combinations of adult exposure ages and ages of onset of leukaemia, and for low doses and low dose rates. Therefore, the models can be applied to assessments of lifetime risk and the detriment.

Ultimately, an improved characterization of leukaemia risk should be feasible by more effectively integrating epidemiological insights with radiobiological research. Understanding of leukaemia development is rapidly evolving. Efforts are ongoing for a more comprehensive characterization of radiation-induced leukaemogenesis within the framework of Adverse Outcome Pathways (AOPs) (Klokov et al. 2022). The potential impact of clonal haematopoiesis of indeterminate potential (CHIP) on leukaemia risk for astronauts was assessed by Werneth et al. (2024). Greenland (1999) proposed that the promotional radiation effect leads to an acceleration of carcinogenesis. The impact of radiation-induced clonal growth on epidemiological risk estimates has been shown in a recent simulation study (Eidemüller et al. 2023). Verification and characterization of radiation-induced CHIP will have important implications for radiation protection through the pathogenic link of CHIP to inflammatory diseases such as atherosclerosis or pulmonary diseases (Cook et al. 2020). The presented risk models can provide an important reference point for such potential developments. This shows the great potential of future research on the interaction between biological mechanisms and improved risk characterization.

## Supplementary Information

Below is the link to the electronic supplementary material.

Supplementary Material 1

## Data Availability

The analysis made use of the ‘lsshempy.csv’ dataset from the Radiation Effects Research Foundation(https://www.rerf.or.jp/en/library/data-en/lsshempy).

## Footnotes

1
https://cran.r-project.org/web/packages/optimx/index.html.
