# Supplementary material for: Leukaemia and lymphoma radiation risk models from atomic bomb survivors (1950–2001) suitable for lifetime risk calculations
Source: Radiat Environ Biophys. 2026 Aug 10;65(4):1029–38. doi: 10.1007/s00411-026-01242-7 (PMC13687226; doi:10.1007/s00411-026-01242-7)
Supplement: Supplementary file 1 [file 411_2026_1242_MOESM1_ESM.docx]

**Leukaemia and lymphoma radiation risk models from atomic bomb survivors suitable for lifetime risk calculations**

**Supplementary Appendix**

**Janine Becker^a^, Felix Heinzl^a^, Maria Schnelzer^a^, Peter Scholz-Kreisel^a^, Munechika Misumi^b^, Hiromi Sugiyama^b^, Kyoji Furukawa^c^, Markus Eidemüller^a^**

^a^ Federal Office for Radiation Protection, Oberschleißheim, Germany

^b^ Radiation Effects Research Foundation, Hiroshima and Nagasaki, Japan

^c^ Biostatistics Center, Kurume University, Kurume, Japan

# Radiation risk models

Exposure from ionizing radiation increases the spontaneous incidence rate $\lambda_{0}$ of leukemia to rate $\lambda$. The radiation risk can be expressed either in form of excess relative risk (ERR) or excess absolute risk (EAR):

$$\lambda=\lambda_{0}\left( 1+\mathrm{ERR} \right)=\lambda_{0}+\mathrm{EAR}$$

$$\lambda_{0}=f(\beta_{b}|s,a,b)$$

$$\left. \begin{matrix} \mathrm{ERR} \\ \mathrm{EAR} \end{matrix} \right\}=r\left( \beta_{r} | D \right) g\left( \beta_{g} | s,a,e, tse \right)$$

(1)

The $\beta_{b}$'s and $\beta_{r}$'s represent the fit parameters of the baseline and radiation risk, respectively. A generic form of the baseline is given by $\lambda_{0}=exp(\beta_{1}+\beta_{2}\cdot ln(a/70)+\beta_{3}\cdot\ln^{2}(a/70)+\beta_{4}\cdot(b-1915)/10)$, the $\beta$'s can also be sex dependent. The variables are defined by:

- *D*: accumulated time-lagged equivalent dose for bone marrow [Gy]
- *a*: attained age [years]
- *e*: age at exposure [years]
- *tse*: time since exposure ($tse=a-e$) [years]
- *b*: birth year
- *s*: indicator for sex:
  - *f*: female ($f=1$ for females, $f=0$ for males)
  - *m*: males ($m=1$ for males, $m=0$ for females)

Details about the fitting procedure can be found in Ulanowski et al. (2020). In the following, the results for the four haematological groups are presented including the covariance matrices.

# Group ALL

The group includes 43 cases in the LSS. The baseline and radiation risk are described as follows, where the baseline rate and EAR are given per 10^4^ person years:

$$\lambda_{0}\left( {10}^{-4} \mathrm{PY}^{-1} \right)=\exp(\beta_{1}+\beta_{2}\ln\frac{a}{70})$$

(2)

$$\mathrm{EAR}_{LNT}\left( {10}^{-4}\mathrm{PY}^{-1} \right)=\beta_{3} D \exp\left[ \beta_{4}\ln\left( \frac{a}{70} \right)+\beta_{5} f \right]$$

(3)

$$\mathrm{EAR}_{QDR}\left( {10}^{-4} \mathrm{PY}^{-1} \right)= \beta_{3} D^{2} \exp\left[ \beta_{4}\ln\left( \frac{a}{70} \right)+\beta_{5} f \right]$$

(4)

The quadratic dose-response model (equation 4) was fitted with an additional factor of 1.12 which represents a correction factor from random errors in the LSS dosimetry system. However, this factor is omitted from the final model (Walsh and Kaiser 2011).

Due to simple form of the baseline, the EAR and ERR formulations of the models are mathematically equivalent under reparameterization. However, the age parameters β₂ and β₄ in the EAR formulation are essentially uncorrelated (correlation coefficient -0.01 for ALL-EAR-LNT), whereas in the corresponding ERR formulation they are highly correlated (correlation coefficient of -0.89). This translates into higher uncertainty in the estimated risk parameters of the ERR model. We therefore selected the EAR models as preferred. The same choice was made in Ulanowski et al. (2020); the present analysis uses a more streamlined baseline. The linear model is also equivalent to that of Hsu et al. (2013), who reported both ERR and EAR formulations.

Table 1: Estimated parameters β_i_ and their standard errors σ_i_ for ALL

|  | ALL-EAR-LNT | |  |  | ALL-EAR-QDR | |
| --- | --- | --- | --- | --- | --- | --- |
| i | β_i_ | σ_i_ |  |  | β_i_ | σ_i_ |
| 1 | -2.350 | 0.264 |  |  | -2.335 | 0.262 |
| 2 | 1.720 | 0.767 |  |  | 1.270 | 0.810 |
| 3 | 0.271 | 0.135 |  |  | 0.169 | 0.087 |
| 4 | -1.785 | 0.370 |  |  | -1.867 | 0.383 |
| 5 | -0.934 | 0.480 |  |  | -0.907 | 0.512 |

Table 2: MMI-weights

|  | ALL-EAR-LNT | ALL-EAR-QDR |
| --- | --- | --- |
| weights | 0.774 | 0.226 |

Table 3: Covariance matrix of β’s for the ALL-EAR-LNT model

|  | β_1_ | β_2_ | β_3_ | β_4_ | β_5_ |
| --- | --- | --- | --- | --- | --- |
| β_1_ | 0.07 | 0.097 | -0.005 | -0.012 | -0.001 |
| β_2_ | 0.097 | 0.589 | 0.001 | -0.002 | 0.006 |
| β_3_ | -0.005 | 0.001 | 0.018 | 0.042 | -0.024 |
| β_4_ | -0.012 | -0.002 | 0.042 | 0.137 | -0.012 |
| β_5_ | -0.001 | 0.006 | -0.024 | -0.012 | 0.231 |

Table 4: Covariance matrix of β’s for the ALL-EAR-QDR model

|  | β_1_ | β_2_ | β_3_ | β_4_ | β_5_ |
| --- | --- | --- | --- | --- | --- |
| β_1_ | 0.069 | 0.121 | -0.002 | -0.01 | 0.006 |
| β_2_ | 0.121 | 0.655 | -0.003 | -0.031 | 0.045 |
| β_3_ | -0.002 | -0.003 | 0.008 | 0.028 | -0.017 |
| β_4_ | -0.01 | -0.031 | 0.028 | 0.146 | -0.016 |
| β_5_ | 0.006 | 0.045 | -0.017 | -0.016 | 0.263 |

# Group AML

The group has 176 cases in the LSS. The dose response is non-linear with an upward curvature. The group consists of two ERR and two EAR models, each with a quadratic dose response (QDR) model, and a two-line-spline (TLS) model. The TLS models are twin pairs to the QDR models. The baseline and radiation risk for the ERR models are given by

$$\lambda_{0}\left( {10}^{-4}\mathrm{PY}^{-1} \right)=\exp\left( \beta_{1} m+\beta_{2} f+\beta_{3} \ln\frac{a}{70}+\beta_{4} ln^{2}\frac{a}{70}+\beta_{5}\frac{b-1915}{10} \right)$$

(5)

$$\mathrm{ERR}_{TLS}=\left\{ \begin{matrix} \beta_{6} D \\ \tau\beta_{6}+\beta_{7}(D-\tau) \end{matrix} \right.\begin{matrix} \mathrm{for} D<\tau\\ \mathrm{for} D\geq\tau\end{matrix}$$

(6)

$$\mathrm{ERR}_{QDR}=\beta_{6} D^{2}$$

(7)

The transition point $\tau$ marks the transition from lower to higher slope in the dose response. The EAR models are given by

$$\lambda_{0}\left( {10}^{-4}\mathrm{PY}^{-1} \right)=\exp\left[ {\beta_{1} m+\beta}_{2} f+{(\beta}_{3} m+\beta_{4} f) \ln\frac{a}{70}+{(\beta}_{5} m+\beta_{6} f) ln^{2}\frac{a}{70}+\beta_{7}\left( \frac{b-1915}{10} \right) \right]$$

(8)

$$\mathrm{EAR}_{TLS}\left( {10}^{-4}\mathrm{PY}^{-1} \right)=\exp\left[ \beta_{8}\ln\frac{a}{70}+\beta_{9}\ln^{2}\left( \frac{a}{70} \right) \right]\times\left\{ \begin{matrix} \beta_{10} D \\ \tau\beta_{10}+\beta_{11}\left( D-\tau\right) \end{matrix} \right.\begin{matrix} \mathrm{for} D<\tau\\ \mathrm{for} D\geq\tau\end{matrix}$$

(9)

$$\mathrm{EAR}_{QDR}\left( {10}^{-4} \mathrm{PY}^{-1} \right)=\exp\left[ \beta_{8}\ln\frac{a}{70}+\beta_{9}\ln^{2}\left( \frac{a}{70} \right) \right] \beta_{10} D^{2}$$

(10)

As for the ALL model, the quadratic ERR and EAR dose response models were fitted with an additional factor of 1.12.

Table 5: Estimated parameters β_i_ and their standard errors σ_i_ for AML

|  | | AML-EAR-TLS | | AML-EAR-QDR | | AML-ERR-TLS | | AML-ERR-QDR | |
| --- | --- | --- | --- | --- | --- | --- | --- | --- | --- |
| i | β_i_ | | σ_i_ | β_i_ | σ_i_ | β_i_ | σ_i_ | β_i_ | σ_i_ |
| 1 | 0.213 | | 0.135 | 0.225 | 0.133 | 0.008 | 0.131 | 0.035 | 0.126 |
| 2 | -0.756 | | 0.145 | -0.721 | 0.138 | -0.668 | 0.126 | -0.638 | 0.120 |
| 3 | 4.235 | | 0.801 | 4.193 | 0.780 | 3.761 | 0.458 | 3.765 | 0.458 |
| 4 | 3.920 | | 0.741 | 3.788 | 0.698 | 1.561 | 0.274 | 1.561 | 0.274 |
| 5 | 1.029 | | 0.793 | 1.048 | 0.760 | 0.128 | 0.059 | 0.127 | 0.059 |
| 6 | 1.680 | | 0.472 | 1.608 | 0.458 | 1.332 | 0.914 | 2.212 | 0.486 |
| 7 | 0.180 | | 0.069 | 0.176 | 0.068 | 7.615 | 1.887 |  |  |
| 8 | 2.511 | | 0.854 | 2.721 | 0.850 |  |  |  |  |
| 9 | 1.375 | | 0.476 | 1.482 | 0.467 |  |  |  |  |
| 10 | 0.798 | | 0.453 | 1.233 | 0.285 |  |  |  |  |
| 11 | 4.243 | | 1.221 |  |  |  |  |  |  |

The transition point $\tau$ for EAR-TLS and ERR-TLS was not fitted automatically, but was sequentially increased in 0.1 Gy steps in the interval [0;1.5], while the rest of the parameters were fitted using the optimisation algorithm in R. Therefore, the parameter $\tau$ has no standard deviation. The final value of $\tau=0.7 \mathrm{Gy}$ was determined by using maximum likelihood.

Table 6: MMI-weights

|  | AML-EAR-TLS | AML-EAR-QDR | AML-ERR-TLS | AML-ERR-QDR |
| --- | --- | --- | --- | --- |
| weights | 0.343 | 0.504 | 0.059 | 0.093 |

Table 7: Covariance matrix of β’s for the AML-EAR-TLS model

|  | β_1_ | β_2_ | β_3_ | β_4_ | β_5_ | β_6_ | β_7_ | β_8_ | β_9_ | β_10_ | β_11_ |
| --- | --- | --- | --- | --- | --- | --- | --- | --- | --- | --- | --- |
| β_1_ | 0.018 | 0.001 | 0.031 | 0.004 | 0.002 | 0.001 | 0.001 | -0.003 | 0 | -0.007 | -0.003 |
| β_2_ | 0.001 | 0.021 | 0.003 | 0.014 | 0.002 | -0.001 | 0.001 | 0.004 | 0.003 | -0.014 | 0.008 |
| β_3_ | 0.031 | 0.003 | 0.641 | 0.121 | 0.482 | 0.042 | 0.027 | -0.049 | -0.026 | 0.024 | -0.047 |
| β_4_ | 0.004 | 0.014 | 0.121 | 0.549 | 0.052 | 0.294 | 0.02 | -0.097 | -0.051 | 0.067 | -0.109 |
| β_5_ | 0.002 | 0.002 | 0.482 | 0.052 | 0.63 | 0.018 | 0.012 | -0.008 | -0.007 | 0.006 | -0.003 |
| β_6_ | 0.001 | -0.001 | 0.042 | 0.294 | 0.018 | 0.223 | 0.007 | -0.041 | -0.024 | 0.032 | -0.045 |
| β_7_ | 0.001 | 0.001 | 0.027 | 0.02 | 0.012 | 0.007 | 0.005 | -0.002 | -0.001 | 0.003 | -0.002 |
| β_8_ | -0.003 | 0.004 | -0.049 | -0.097 | -0.008 | -0.041 | -0.002 | 0.73 | 0.377 | -0.013 | 0.543 |
| β_9_ | 0 | 0.003 | -0.026 | -0.051 | -0.007 | -0.024 | -0.001 | 0.377 | 0.226 | -0.022 | 0.225 |
| β_10_ | -0.007 | -0.014 | 0.024 | 0.067 | 0.006 | 0.032 | 0.003 | -0.013 | -0.022 | 0.206 | -0.139 |
| β_11_ | -0.003 | 0.008 | -0.047 | -0.109 | -0.003 | -0.045 | -0.002 | 0.543 | 0.225 | -0.139 | 1.491 |

Table 8: Covariance matrix of β’s for the AML-EAR-QDR model

|  | β_1_ | β_2_ | β_3_ | β_4_ | β_5_ | β_6_ | β_7_ | β_8_ | β_9_ | β_10_ |
| --- | --- | --- | --- | --- | --- | --- | --- | --- | --- | --- |
| β_1_ | 0.018 | 0 | 0.032 | 0.006 | 0.003 | 0.002 | 0.002 | -0.004 | -0.001 | -0.003 |
| β_2_ | 0 | 0.019 | 0.005 | 0.02 | 0.002 | 0.002 | 0.001 | -0.001 | 0 | -0.003 |
| β_3_ | 0.032 | 0.005 | 0.609 | 0.104 | 0.456 | 0.035 | 0.025 | -0.025 | -0.014 | 0.001 |
| β_4_ | 0.006 | 0.02 | 0.104 | 0.487 | 0.047 | 0.266 | 0.018 | -0.049 | -0.025 | 0.003 |
| β_5_ | 0.003 | 0.002 | 0.456 | 0.047 | 0.578 | 0.016 | 0.012 | -0.007 | -0.006 | 0.002 |
| β_6_ | 0.002 | 0.002 | 0.035 | 0.266 | 0.016 | 0.21 | 0.006 | -0.018 | -0.012 | 0.003 |
| β_7_ | 0.002 | 0.001 | 0.025 | 0.018 | 0.012 | 0.006 | 0.005 | 0 | 0 | 0.001 |
| β_8_ | -0.004 | -0.001 | -0.025 | -0.049 | -0.007 | -0.018 | 0 | 0.723 | 0.367 | 0.115 |
| β_9_ | -0.001 | 0 | -0.014 | -0.025 | -0.006 | -0.012 | 0 | 0.367 | 0.218 | 0.041 |
| β_10_ | -0.003 | -0.003 | 0.001 | 0.003 | 0.002 | 0.003 | 0.001 | 0.115 | 0.041 | 0.081 |

Table 9: Covariance matrix of β’s for the AML-ERR-TLS model

|  | β_1_ | β_2_ | β_3_ | β_4_ | β_5_ | β_6_ | β_7_ |
| --- | --- | --- | --- | --- | --- | --- | --- |
| β_1_ | 0.017 | 0.005 | 0.018 | 0.005 | 0.001 | -0.04 | -0.061 |
| β_2_ | 0.005 | 0.016 | 0.011 | 0.003 | 0.001 | -0.042 | -0.046 |
| β_3_ | 0.018 | 0.011 | 0.209 | 0.107 | 0.015 | 0.002 | 0.037 |
| β_4_ | 0.005 | 0.003 | 0.107 | 0.075 | 0.005 | 0.001 | 0.02 |
| β_5_ | 0.001 | 0.001 | 0.015 | 0.005 | 0.003 | 0.001 | -0.001 |
| β_6_ | -0.04 | -0.042 | 0.002 | 0.001 | 0.001 | 0.836 | -0.271 |
| β_7_ | -0.061 | -0.046 | 0.037 | 0.02 | -0.001 | -0.271 | 3.561 |

Table 10: Covariance matrix of β’s for the AML-ERR-QDR model

|  | β_1_ | β_2_ | β_3_ | β_4_ | β_5_ | β_6_ |
| --- | --- | --- | --- | --- | --- | --- |
| β_1_ | 0.016 | 0.003 | 0.018 | 0.005 | 0.001 | -0.023 |
| β_2_ | 0.003 | 0.014 | 0.011 | 0.003 | 0.001 | -0.019 |
| β_3_ | 0.018 | 0.011 | 0.21 | 0.107 | 0.015 | 0.01 |
| β_4_ | 0.005 | 0.003 | 0.107 | 0.075 | 0.005 | 0.005 |
| β_5_ | 0.001 | 0.001 | 0.015 | 0.005 | 0.003 | 0 |
| β_6_ | -0.023 | -0.019 | 0.01 | 0.005 | 0 | 0.236 |

# Group CML

The CML group includes 75 cases in the LSS. Radiation risk is represented by three ERR models, each is given a MMI weight of 1/3. Two models depend strongly on time since exposure *tse*. In the step model, the parameter fit for periods of 40 years or more since exposure converges to zero.

In a plausibility check we varied the weights to ½ for the two-step model and 1/4 for the other two models, and also applied weightings based on AIC. For special exposure scenarios this could lead to relatively large changes in the assigned share for small changes in time since exposure. For use in ProZES an equal weighting of 1/3 for each model was considered most appropriate.

$$\lambda_{0}\left( {10}^{-4}\mathrm{PY}^{-1} \right)=\exp\left[ \beta_{1}+{(\beta_{2} m+\beta}_{3} f) \ln\frac{a}{70} \right]$$

(11)

$$\mathrm{ERR}_{const}=\beta_{4} D$$

(12)

$${\mathrm{ERR}_{step}=\left\{ \begin{matrix} e^{\beta_{4}} D & 0\leq tse<10 \\ e^{\beta_{5}} D & 10\leq tse<40 \\ 0 D & 40\leq tse \end{matrix} \right.}$$

(13)

$$\mathrm{ERR}_{exp}=\beta_{4} D e^{\beta_{5} tse}$$

(14)

Table 11: Estimated parameters β_i_ and their standard errors σ_i_ for CML

|  | CML-ERR-const | |  | CML-ERR-step | |  | CML-ERR-exp | |
| --- | --- | --- | --- | --- | --- | --- | --- | --- |
| i | β_i_ | σ_i_ |  | β_i_ | σ_i_ |  | β_i_ | σ_i_ |
| 1 | -1.915 | 0.196 |  | -1.756 | 0.180 |  | -1.755 | 0.182 |
| 2 | -0.030 | 0.282 |  | 0.478 | 0.294 |  | 0.510 | 0.298 |
| 3 | 1.948 | 0.56 |  | 2.521 | 0.574 |  | 2.585 | 0.578 |
| 4 | 8.222 | 2.438 |  | 3.706 | 0.314 |  | 74.228 | 31.922 |
| 5 |  |  |  | 1.733 | 0.397 |  | -0.108 | 0.027 |

Table 12: MMI-weights

|  | CML-ERR-const | CML-ERR-step | CML-ERR-exp |
| --- | --- | --- | --- |
| weights | 1/3 | 1/3 | 1/3 |

Table 13: Covariance matrix of β’s for the CML-ERR-const model

|  | β_1_ | β_2_ | β_3_ | β_4_ |
| --- | --- | --- | --- | --- |
| β_1_ | 0.038 | 0.021 | 0.029 | -0.322 |
| β_2_ | 0.021 | 0.079 | 0.032 | 0.009 |
| β_3_ | 0.029 | 0.032 | 0.313 | 0.031 |
| β_4_ | -0.322 | 0.009 | 0.031 | 5.942 |

Table 14: Covariance matrix of β’s for the CML-ERR-exp model

|  | β_1_ | β_2_ | β_3_ | β_4_ | β_5_ |
| --- | --- | --- | --- | --- | --- |
| β_1_ | 0.033 | 0.016 | 0.022 | -1.182 | -0.001 |
| β_2_ | 0.016 | 0.089 | 0.044 | 2.908 | -0.001 |
| β_3_ | 0.022 | 0.044 | 0.334 | 3.462 | -0.001 |
| β_4_ | -1.182 | 2.908 | 3.462 | 1019.018 | -0.625 |
| β_5_ | -0.001 | -0.001 | -0.001 | -0.625 | 0.001 |

Table 15: Covariance matrix of β’s for the CML-ERR-step model

|  | β_1_ | β_2_ | β_3_ | β_4_ | β_5_ |
| --- | --- | --- | --- | --- | --- |
| β_1_ | 0.032 | 0.016 | 0.022 | -0.024 | -0.039 |
| β_2_ | 0.016 | 0.086 | 0.041 | 0.028 | 0.006 |
| β_3_ | 0.022 | 0.041 | 0.329 | 0.034 | 0.018 |
| β_4_ | -0.024 | 0.028 | 0.034 | 0.099 | 0.046 |
| β_5_ | -0.039 | 0.006 | 0.018 | 0.046 | 0.157 |

# Group LYM

The group includes 449 cases in the LSS, 402 of these cases are non-Hodgkin lymphoma. All risk models use the same parametric form for the baseline.

$$\lambda_{0}\left( {10}^{-4}\mathrm{PY}^{-1} \right)=\exp\left[ \beta_{1} m+\beta_{2} f+ \beta_{3}\ln\frac{a}{70}+\beta_{4} m ln^{2}\frac{a}{70}+\beta_{5}\left( \frac{b-1915}{10} \right) \right]$$

(15)

$$\mathrm{EAR}\left( {10}^{-4} \mathrm{PY}^{-1} \right)= \beta_{6} D$$

(16)

$$ERR= \beta_{6} D$$

(17)

The models (16) and (17) were fitted for all LSS members as well as for males only and for females only. Radiation risks for females were nearly zero ($\beta_{6}$ in model (16) and model (17)). Therefore, only the models for males only (with total of 50% MMI-weight), and for all LSS members were used in the following (with total of 50% MMI-weight).

Table 16: Estimated parameters β_i_ and their standard errors σ_i_ for LYM

|  | LYM-EAR-LNT-joint | |  | LYM-EAR-LNT-male | |  | LYM-ERR-LNT-joint | |  | LYM-ERR-LNT-male | |
| --- | --- | --- | --- | --- | --- | --- | --- | --- | --- | --- | --- |
| i | β_i_ | σ_i_ |  | β_i_ | σ_i_ |  | β_i_ | σ_i_ |  | β_i_ | σ_i_ |
| 1 | 1.257 | 0.076 |  | 1.241 | 0.078 |  | 1.229 | 0.077 |  | 1.198 | 0.082 |
| 2 | 0.761 | 0.066 |  | 0.769 | 0.066 |  | 0.742 | 0.068 |  | 0.768 | 0.066 |
| 3 | 4.538 | 0.299 |  | 4.528 | 0.301 |  | 4.513 | 0.292 |  | 4.512 | 0.292 |
| 4 | 1.181 | 0.366 |  | 1.008 | 0.430 |  | 1.290 | 0.308 |  | 1.288 | 0.309 |
| 5 | 0.250 | 0.037 |  | 0.249 | 0.037 |  | 0.248 | 0.037 |  | 0.248 | 0.037 |
| 6 | 0.134 | 0.140 |  | 0.626 | 0.355 |  | 0.294 | 0.205 |  | 0.608 | 0.343 |

Table 17: MMI-weights

|  | LYM-EAR-LNT-joint | LYM-EAR-LNT-male | LYM-ERR-LNT-joint | LYM-ERR-LNT-male |
| --- | --- | --- | --- | --- |
| weights | 0.171 | 0.31 | 0.329 | 0.19 |

Table 18: Covariance matrix of β’s for the LYM-EAR-LNT-joint model

|  | β_1_ | β_2_ | β_3_ | β_4_ | β_5_ | β_6_ |
| --- | --- | --- | --- | --- | --- | --- |
| β_1_ | 0.006 | 0 | 0.004 | -0.004 | 0 | 0 |
| β_2_ | 0 | 0.004 | 0.003 | 0.003 | 0 | -0.001 |
| β_3_ | 0.004 | 0.003 | 0.089 | 0.06 | 0.006 | 0.003 |
| β_4_ | -0.004 | 0.003 | 0.06 | 0.134 | 0.002 | -0.01 |
| β_5_ | 0 | 0 | 0.006 | 0.002 | 0.001 | 0 |
| β_6_ | 0 | -0.001 | 0.003 | -0.010 | 0 | 0.020 |

Table 19: Covariance matrix of β’s for the LYM-EAR-LNT-male model

|  | β_1_ | β_2_ | β_3_ | β_4_ | β_5_ | β_6_ |
| --- | --- | --- | --- | --- | --- | --- |
| β_1_ | 0.006 | 0 | 0.004 | -0.006 | 0 | -0.004 |
| β_2_ | 0 | 0.004 | 0.003 | 0.003 | 0 | 0 |
| β_3_ | 0.004 | 0.003 | 0.09 | 0.068 | 0.006 | 0.003 |
| β_4_ | -0.006 | 0.003 | 0.068 | 0.185 | 0.002 | -0.023 |
| β_5_ | 0 | 0 | 0.006 | 0.002 | 0.001 | 0 |
| β_6_ | -0.004 | 0 | 0.003 | -0.023 | 0 | 0.126 |

Table 20: Covariance matrix of β’s for the LYM-ERR-LNT-joint model

|  | β_1_ | β_2_ | β_3_ | β_4_ | β_5_ | β_6_ |
| --- | --- | --- | --- | --- | --- | --- |
| β_1_ | 0.006 | 0.001 | 0.004 | -0.003 | 0 | -0.004 |
| β_2_ | 0.001 | 0.005 | 0.003 | 0.002 | 0 | -0.004 |
| β_3_ | 0.004 | 0.003 | 0.085 | 0.055 | 0.006 | 0.001 |
| β_4_ | -0.003 | 0.002 | 0.055 | 0.095 | 0.002 | 0.001 |
| β_5_ | 0 | 0 | 0.006 | 0.002 | 0.001 | 0 |
| β_6_ | -0.004 | -0.004 | 0.001 | 0.001 | 0 | 0.042 |

Table 21: Covariance matrix of β’s for the LYM-ERR-LNT-male model

|  | β_1_ | β_2_ | β_3_ | β_4_ | β_5_ | β_6_ |
| --- | --- | --- | --- | --- | --- | --- |
| β_1_ | 0.007 | 0 | 0.004 | -0.003 | 0 | -0.011 |
| β_2_ | 0 | 0.004 | 0.003 | 0.002 | 0 | 0 |
| β_3_ | 0.004 | 0.003 | 0.085 | 0.055 | 0.006 | 0.001 |
| β_4_ | -0.003 | 0.002 | 0.055 | 0.095 | 0.002 | 0.001 |
| β_5_ | 0 | 0 | 0.006 | 0.002 | 0.001 | 0 |
| β_6_ | -0.011 | 0 | 0.001 | 0.001 | 0 | 0.118 |

**References**

Hsu WL, Preston DL, Soda M, Sugiyama H, Funamoto S, Kodama K, Kimura A, Kamada N, Dohy H, Tomonaga M, Iwanaga M, Miyazaki Y, Cullings HM, Suyama A, Ozasa K, Shore RE, Mabuchi K (2013) The incidence of leukemia, lymphoma and multiple myeloma among atomic bomb survivors: 1950-2001. Radiat Res 179 (3):361-382. doi:10.1667/RR2892.1

Ulanowski A, Shemiakina E, Guthlin D, Becker J, Preston D, Apostoaei AI, Hoffman FO, Jacob P, Kaiser JC, Eidemuller M (2020) ProZES: the methodology and software tool for assessment of assigned share of radiation in probability of cancer occurrence. Radiat Environ Biophys 59 (4):601-629. doi:10.1007/s00411-020-00866-7

Walsh L, Kaiser JC (2011) Multi-model inference of adult and childhood leukaemia excess relative risks based on the Japanese A-bomb survivors mortality data (1950-2000). Radiat Environ Biophys 50 (1):21-35. doi:10.1007/s00411-010-0337-6
